# A cell design for correlative hard X-ray nanoprobe and electron microscopy studies of catalysts under *in situ* conditions

**DOI:** 10.1107/S1600577521013576

**Published:** 2022-02-15

**Authors:** Julia E. Parker, Miguel Gomez-Gonzalez, Yolanda Van Lishout, Husn Islam, Desiree Duran Martin, Dogan Ozkaya, Paul D. Quinn, Manfred E. Schuster

**Affiliations:** a Diamond Light Source, Harwell Science and Innovation Campus, Didcot, Oxfordshire OX11 0DE, United Kingdom; b Johnson Matthey Technology Centre, Johnson Matthey, Blounts Court, Sonning Common, Berkshire RG4 9NH, United Kingdom

**Keywords:** *in situ*, sample environments, multi-length scales, X-ray nanoprobes, transmission electron microscopy (TEM), synchrotrons, micro-electro-mechanical systems (MEMS)

## Abstract

To improve the understanding of catalysts, and ultimately the ability to design better materials, it is crucial to study them during their catalytic active states. Using *in situ* or *operando* conditions allows insights into structure–property relationships, which might not be observable by *ex situ* characterization. Spatially resolved X-ray fluorescence, X-ray diffraction and X-ray absorption near-edge spectroscopy are powerful tools to determine structural and electronic properties, and the spatial resolutions now achievable at hard X-ray nanoprobe beamlines make them an ideal complement to high-resolution transmission electron microscopy studies in a multi-length-scale analysis approach. The development of a system to enable the use of a commercially available gas-cell chip assembly within an X-ray nanoprobe beamline is reported here. The novel *in situ* capability is demonstrated by an investigation of the redox behaviour of supported Pt nanoparticles on ceria under typical lean and rich diesel-exhaust conditions; however, the system has broader application to a wide range of solid–gas reactions. In addition the setup allows complimentary *in situ* transmission electron microscopy and X-ray nanoprobe studies under identical conditions, with the major advantage compared with other systems that the exact same cell can be used and easily transferred between instruments. This offers the exciting possibility of studying the same particles under identical conditions (gas flow, pressure, temperature) using multiple techniques.

## Abstract 2

The development of an *in situ* gas/heating cell for X-ray nanoprobe studies is presented. The capabilities are demonstrated by an investigation of the redox behaviour of supported Pt nanoparticles on ceria.

## 1. Introduction

Revealing the structure–activity relationship in catalysis is crucial to understanding the role of the catalyst during chemical processes and an essential step towards the ‘design of catalysts’. It is widely accepted that a necessary step for this is studying the catalyst during relevant working conditions (pressure/temperature). In order to achieve this, *in situ* or *operando* (*in situ* linked to catalytic activity) studies are of crucial importance (Topsøe, 2003 ▸; Arrigo *et al.*, 2013 ▸; Han *et al.*, 2015 ▸; Streibel *et al.*, 2018 ▸; Weckhuysen, 2002 ▸)

One of the biggest challenges in catalysis research is linking macro-scale and micro-scale properties with highly spatially resolved techniques. While the former is crucial to provide a representative analysis of a catalyst, the latter is crucial to resolve the often-complex links between formulation and operation. Multi-scale and multi-technique approaches offer a solution, and the combination of a highly spatially resolved technique, such as aberration-corrected transmission electron microscopy (ACTEM) which can achieve sub-angstrom resolution (with the limitation of only probing a small area), with synchrotron-based nano-imaging provides a mechanism to bridge length scales between active sites and microstructure. Developments of hard X-ray focusing optics and ptychography over recent years have led to increased spatial resolution of tens of nanometres over fields of view of ∼100 µm (Nazaretski *et al.*, 2014 ▸; Martínez-Criado *et al.*, 2016 ▸; Huang *et al.*, 2013 ▸; Dierolf *et al.*, 2010 ▸) bridging the resolution gap to electron microscopy studies, enabling a multi-scale approach with an overlap of the sampling volume between electrons (ACTEM) and X-rays.

To achieve this multi-scale approach, sample environments which replicate the operating conditions are needed, as well as an ability to move these sample environments between instruments. While initial *in situ* microscopy studies (Hansen, 2001 ▸) were limited by the achievable pressure in so-called environmental transmission electron microscopes, which are in the millibar range, the more recent advent of micro-electro-mechanical systems (MEMS) based *in situ* cells has enabled atomic resolution TEM studies at more relevant catalytic conditions, such as reaction cell pressures of up to 1 bar and temperatures of up to 1000°C (Lang *et al.*, 2019 ▸). X-ray sample environment developments have also progressed in order to accommodate the restrictions imposed by nanoscale scanning beamlines (limited space, maximum weight, thermal stability *etc*.) (Kelly *et al.*, 2013 ▸; Prabu *et al.*, 2018 ▸; Bozzini *et al.*, 2017 ▸). Recent developments towards *in situ* microscopy and multi-scale studies (Baier *et al.*, 2016 ▸; van Ravenhorst *et al.*, 2019 ▸; Gonzalez-Jimenez *et al.*, 2012 ▸) have focused on the design of a bespoke MEMS-based *in situ* X-ray cell to complement *in situ* TEM experiments. Our work focuses on adapting a transmission electron microscope holder for dual use to enable *in situ* experiments in both a transmission electron microscope and scanning-probe beamline.

This approach has several advantages over designing a new cell. Firstly, using the same cell means both experiments will happen under identical flow conditions, temperature ramps, pressure conditions and sample confinement. This will make complementary experiments truly comparable. Secondly, and equally important for ease of use, using the same cell allows for well defined registration between the instruments to correlate *in situ* TEM with *in situ* X-ray fluorescence (XRF), X-ray diffraction (XRD) and X-ray absorption near-edge spectroscopy (XANES) on the same region or particle of the sample. This is particularly important with the narrower field of view of TEM. Fam *et al.* (2019 ▸) followed a similar approach of using TEM-based MEMS chips, with the significant difference that the MEMS chip needs to be removed from the *in situ* X-ray cell to be subsequently analysed in the transmission electron microscope, which can lead to rotational mis-registration, and the process of disassembly needs to be undertaken with care to avoid damaging the fragile silicon nitride membranes of the chips. The additional, and in some cases crucial, advantage of directly using the transmission electron microscope *in situ* cell is a possible transfer of the cell between nanoprobe beamline and electron microscope without changing the gas environment, and hence the sample state, enabling a true correlative and multi-scale study.

The system presented has been implemented at I14, the hard X-ray nanoprobe beamline (Quinn *et al.*, 2021*a* ▸), and the electron physical science imaging centre (ePSIC) at Diamond Light Source. The setup allows complimentary *in situ* TEM and X-ray microscopy under identical conditions, with the major advantage compared with other systems that the exact same cell is used in both and therefore the same particles can be studied under identical conditions (gas flow, pressure, temperature). This work will present the modifications made to the *in situ* transmission electron microscope cell to allow its use on the nanoprobe beamline, and operational performance during a study of Pt-supported catalyst samples by means of X-ray nanoprobe XRF and XANES.

## 2. Design of the *in situ* cell

**Figure 1:**
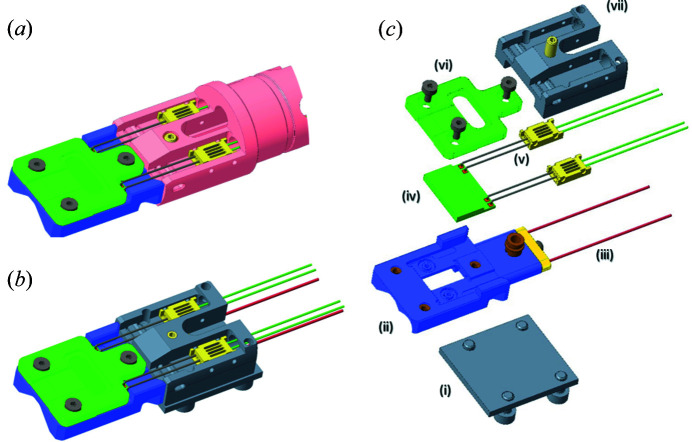
Schematic drawing of the DENSsolutions climate holder tip as assembled in (*a*) the electron microscope holder and (*b*) the beamline adaptor. The exploded view (*c*) shows (i) the backplate of the beamline adaptor piece, (ii) the tip bottom piece with (iii) attached gas tubing, (iv) the nanoreactor chip, (v) needle heating contacts and cables, (vi) the tip top piece to secure and seal the nanoreactor chip, and (vii) the main piece of the beamline adaptor which, when fully assembled, houses the needle contacts.

The *in situ* solution developed here consists of an adaption of a commercial transmission electron microscope holder (climate holder, DENSsolutions, Delft, Netherlands), which is a dedicated *in situ* holder to enable the study of gas–solid interactions at temperatures up to 1000°C and cell pressures up to 1 bar within the vacuum of a transmission electron microscope. The details of the cell parts are illustrated in Fig. 1 ▸, showing the assembly in the electron microscope holder and in the beamline configuration.

The holder carries the nanoreactor chips consisting of two patterned silicon nitride membranes separated by a rubber ring (Creemer *et al.*, 2008 ▸; Perez-Garcia *et al.*, 2016 ▸) (Fig. 1 ▸). The top membrane defines a channel for the flow of gas and the bottom membrane has a patterned micro-heater spiral and apertures for the gas inlet and outlet. Both membranes also have a recess for the rubber O-ring. The top and bottom chips have a thickness of 400 nm of silicon nitride, across an 800 µm × 800 µm window area. The chips contain several thinned areas of 20 nm silicon nitride thickness and 6 µm diameter to allow for electron transmission. The assembly of the cell requires an alignment of the transmission windows, O-ring and the channel of the top membrane with the inlet and outlet of the bottom membrane. The current from the temperature-controller box (power supply) is delivered through spring-loaded needle contacts to patterned pad contacts on the MEMS chip. The temperature of the heating spiral is monitored and controlled using the temperature-dependent resistance of the micro-heater, and manufacturing variations are dealt with *via* a supplied chip-specific calibration which is input to the controller (van Omme *et al.*, 2018 ▸).

**Figure 2:**
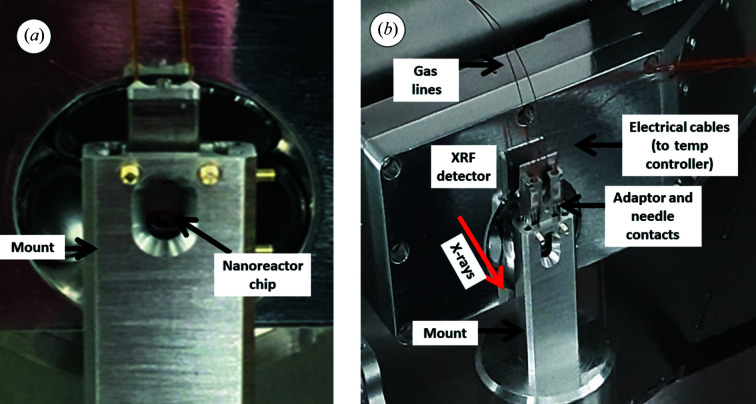
(*a*) The cell tip housing the nanoreactor chip (without adaptor piece) aligned in the beamline mount. (*b*) The cell tip and adaptor piece fully assembled and connected in the beamline mount on beamline I14.

The modular design of this *in situ* holder enables the removal of the tip, which holds the nanoreactor chips, from the TEM holder (Fig. 1 ▸), whilst keeping the gas-flow tubing in place and connected to the gas supply system. In order for the tip to then be mounted onto the beamline, an adaptor piece containing the needle heating contacts is attached to remake the electrical connections between the temperature controller and the chip (Fig. 1 ▸). The assembly can then be aligned and fixed in a mount on the beamline sample stages (Fig. 2 ▸)

The mount was designed specifically for the I14 stages and working-distance constraints. The cell along the beam direction is <10 µm thick so would be suitable for use across soft and hard X-ray nanofocusing instruments; for example, the scanning X-ray microscopy beamline, I08, at Diamond. For lower-energy X-rays the X-ray transmission of the cell must be carefully considered, and the thinned transmission windows designed for TEM imaging can be utilized. As a comparison, for the energy range of the I14 nanoprobe beamline (5–20 keV) the transmission is >96% for the full thickness of the silicon nitride windows (800 nm total thickness) and a typical gas layer, this would reduce to ∼70% for I08 (250–4200 eV) over the thinned areas (40 nm total thickness).

The identical gas and heater connections allow for accurate and consistent temperature and gas control when mounted on the beamline as well as the electron microscope. As the exact same system is used both for *in situ* TEM and synchrotron experiments, identical experimental conditions can be achieved which allows a direct *in situ* correlation between these two techniques.

The I14 beamline (Quinn *et al.*, 2021*a* ▸) uses a back-scatter XRF geometry allowing for unobstructed measurement of the XRF signal from the cell in the mount, and the cell mount has a sufficient aperture at the rear to allow for transmission XRD measurements with a ±40° cone angle. The overall cell assembly is thin enough to sit within the depth of focus of the beam and allows for transmission imaging techniques such as ptychography to also be employed.

The sample dimensions perpendicular to the probe are limited by the size of the heating area on the MEMS chips, 500 µm × 500 µm, and along the beam axis by the distance between top and bottom chip, which is 5–8 µm, depending on the relative pressure difference between the interior and exterior of the cell. For instances when the cell will be used to carry out complementary *in situ* synchrotron and TEM experiments, the sample dimension in the *z* direction should be limited to the hundreds of nanometres range due to the limited penetration depth of the electron beam. This thickness constraint can be relaxed for X-ray only experiments. Drop casting, focused ion beam sections or microtome slices can be used within the cell depending on the experiment and sample preparation needs.

## 3. Cell characterization

The operation of the cell on a beamline and in a transmission electron microscope requires both changes in geometry (transmission electron microscope – horizontal mounting, beamline – vertical mounting) and external atmosphere (transmission electron microscope – vacuum, beamline – air), but importantly the cell assembly and gas lines remain in place to allow for dual use. Characterization of the heating and gas-flow performance of the cell was undertaken to understand conditions during normal operation.

### 3.1. Gas-flow and heat-distribution simulations

*COMSOL* simulations (COMSOL Inc., Stockholm, Sweden, https://www.comsol.com) were carried out to determine the gas properties inside the beam well (above the heater area). A simplified geometry of the cell was used, removing any unnecessary details in order to optimize mesh creation and produce a 3D representation of the cell. The gas flow and temperature inside the cell were modelled using coupled computational fluid dynamics (CFD) and heat-transfer modules, respectively. The CFD model was validated by comparing the estimated mass flow and overall pressure drop of the cell with experimental data obtained by flowing He through the cell at room temperature, at various inlet pressures, measuring the outlet pressure and mass-flow rate.

**Figure 3:**
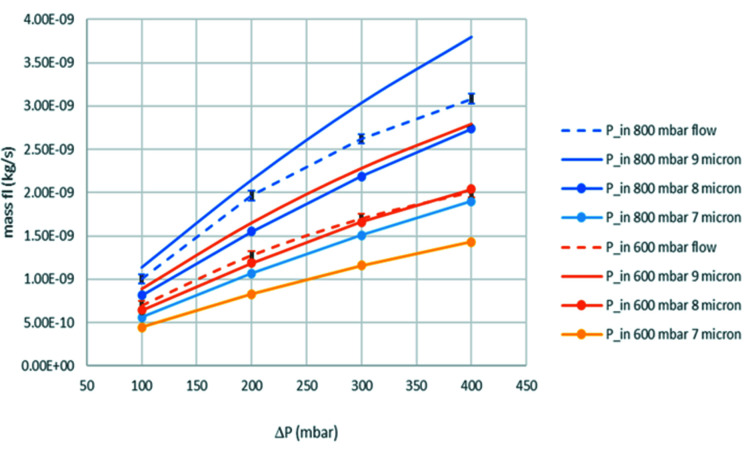
Experimentally measured mass flow (dotted lines) and simulated pressure drop (solid lines) for a range of gap sizes and inlet pressures of 800 and 600 mbar.

Fig. 3 ▸ shows the experimentally measured mass flow (dotted lines), for inlet pressures of 800 mbar (blue) and 600 mbar (orange) and various outlet pressures, given the pressure difference as indicated on the *x* axis. In addition, the graph shows the simulated pressure drop for a 7, 8 and 9 µm gap width between the cell halves’ surface (solid lines), for the same inlet and outlet pressures as the experimental values. The graphs reveal the best match between the simulation and experimental curves to be the 600 mbar inlet pressure for the 8 µm gap width, while the fit for the 800 mbar inlet pressure between simulated and experimental values is less good (the experimental curve lies between the 8 and 9 µm-gap-width simulation curves), suggesting that when using higher pressure the gap width between the two cell halves may increase. Based on these results we conclude that simulating an 8 µm gap width between the upper and lower cell halves, with the O-ring in place, leads to the best match for pressure drop and mass flow with the experimental data collected.

Further considerations were given to the potential bulging of the SiNx membrane windows due to thermal expansion during the heating, as well as due to pressure differential between the cell interior and exterior. The interior cell pressure can be up to 1 bar, whereas the exterior pressure, when operating inside the TEM, can be as low as of the order of 10^−5^ Pa. *COMSOL* simulations showed that, while the increased gap width inside the beam well due to this bulging leads to a locally reduced pressure drop, the pressure drop across the whole cell remains unchanged.

**Figure 4:**
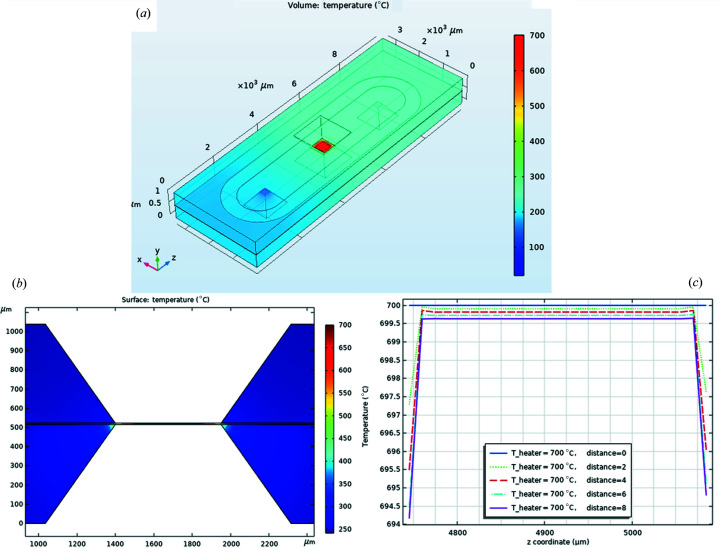
(*a*) A temperature profile for the insulated chip from *COMSOL* simulations. (*b*) An *x*–*y* axis slice through the heater-well area and (*c*) temperature as a function of distance from the heater-chip surface. The simulations were carried out outside the transmission electron microscope using He gas and a mass flow of 2.64 × 10^−9^ kg s^−1^. The ambient temperature was 20°C. A range of temperatures for the heater including 20, 200, 400 and 700°C are compared.

The heat-transfer model describes the heat conduction in the Si wafer halves of the cell, including heat losses via natural convection, when operated outside the transmission electron microscope, as well as radiation. The heater is represented as a constant temperature boundary condition in the beam well by a square surface having 500 µm-length sides. The gas enters the cell at room temperature, being pre-heated to some extent by the warm surfaces of the Si cell halves, and is further heated to the final temperature inside the beam well before cooling as it moves along to the exit of the cell [Fig. 4 ▸(*a*)]. Steady-state simulations were carried out for various gas mass-flow rates at different inlet and outlet pressures and heater set-point temperature, for operation inside as well as outside the transmission electron microscope. The simulations presented in Fig. 4 ▸ are for the cell outside the transmission electron microscope using He gas with a mass flow of 2.64 × 10^−9^ kg s^−1^. The ambient temperature was 20°C. A range of temperatures for the heater including 20, 200, 400 and 700°C are compared. The values for flow rates, inlet and outlet pressures as used in the simulations were compared with experimental data. The gas temperature inside the beam well [Fig. 4 ▸(*a*)] is the same as the heater set-point temperature for all the various operating conditions simulated. The simulations show that the temperature above the heater inside the beam well, between the upper and lower half cell beam membranes, is uniform for several micrometres [Fig. 4 ▸(*b*)]. This is very important as it allows us to exclude any cooling effect of the gas on the catalyst surface. Such a cooling would make a direct interpretation of any data more complicated as the temperature set point would not be the ‘real’ temperature of the catalyst and therefore any attempt to carry out a catalytic structure–activity correlation would be inaccurate. Notably, with increased *z* distance from the heater chip, a slight decrease in the calculated temperature is observed on the outside of the heater chip [Fig. 4 ▸(*c*)]; however, the simulation indicates that the central 300 µm of the heater chip maintains the set temperature. As the catalyst particles are usually deposited on the central area of the chip, located on the thinned window areas for TEM imaging, the slight drop of temperature on the ‘external parts’ of the heater should not have any detrimental effect.

### 3.2. Operational properties

As indicated by the simulations, while the temperature of the spiral is monitored and controlled using the temperature-dependent resistance of the micro-heater, this is an average and the uniformity of this temperature may vary over the chip. In order to validate and quantify the thermal performance of the cell in the X-ray beamline geometry, the thermal lattice expansion of platinum particles was determined as a function of cell temperature using XRD measurements.

**Figure 5:**
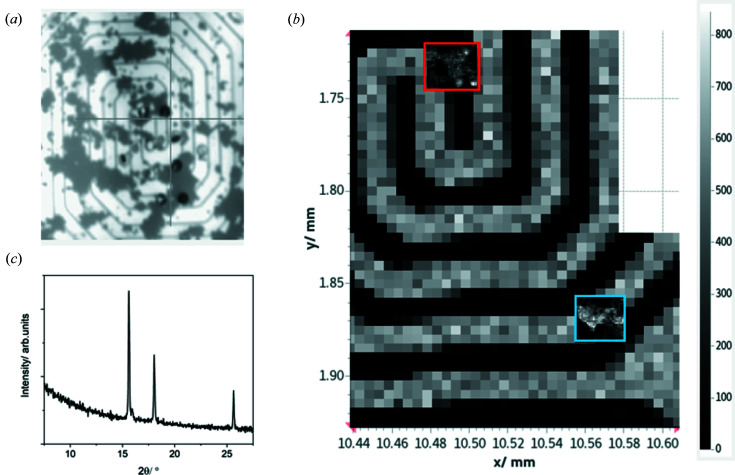
The locations of the centre and edge areas where XRD data were collected as a function of temperature. (*a*) An optical microscope image of Pt powder on the bottom chip showing the locations of the thinned areas for electron microscope measurements. (*b*) A Mo map of the heater spiral with inset Pt maps from (red box) centre and (blue box) edge areas. (*c*) An example XRD pattern from the mapped area.

**Figure 6:**
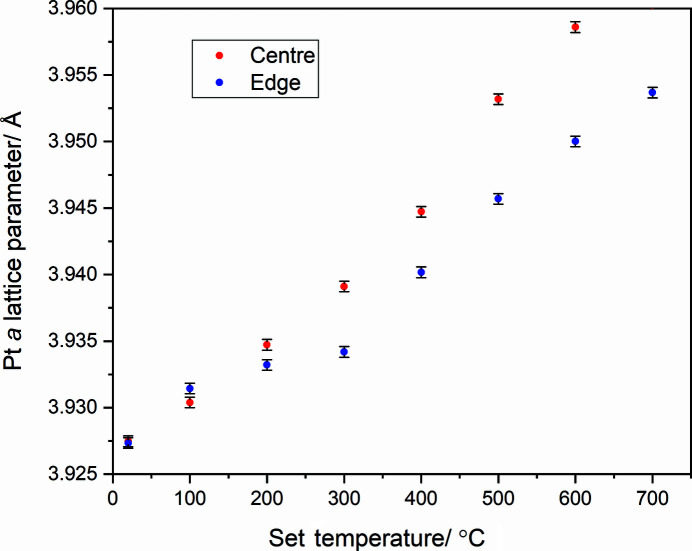
Pt *a* lattice parameter of nanoparticles in the centre (red) and edge (blue) regions as a function of the set temperature of the sample cell.

Platinum powder was dispersed onto a heater chip and mounted in the cell on the nanoprobe beamline, with the gas lines open to air. XRD mapping data were acquired at 20 keV in transmission geometry. Data were collected using an Excalibur detector (Marchal *et al.*, 2013 ▸) at a sample–detector distance of 240 mm, calibrated using a CeO_2_ standard (NIST SRM674b). XRD maps were collected from ∼2.5 µm × 2.5 µm regions selected to be near the centre of the heater spiral and at the edge (Fig. 5 ▸). The 2D images were azimuthally integrated using *DAWN* (Filik *et al.*, 2017 ▸) and the diffraction maps were summed across each region to give an aggregate powder pattern at each temperature (<700°C), which was refined [*TOPAS* (Coelho, 2018 ▸)] to extract the platinum lattice parameter. Fig. 6 ▸ shows that at low temperatures (<200°C) the lattice parameters, and hence temperatures, of the two regions are in agreement; however, for hotter temperatures the edge region lags behind the centre. The edge region was selected to be distant from the electron microscopy thinned viewing areas [Fig. 5 ▸(*a*)], but it is on the edge of the central 300 µm region suggested by the *COMSOL* simulations to maintain the set temperature. This difference may arise due to difference in the gas-flow conditions between the simulations and experiment. This temperature difference must however be considered when regions of interest are selected for X-ray microscopy measurements. These variations across the MEMS chip have been observed in recent optical measurements of the same MEMS cells (van Ravenhorst *et al.*, 2019 ▸). Diffraction-based temperature monitoring provides a mechanism for thermal measurement within the existing multimodal infrastructure of the beamline without the need for additional equipment or modification of the endstation and is also possible to apply to TEM (Niekiel *et al.*, 2017 ▸).

## 4. *In situ* hard X-ray nanoprobe study of model Pt NOx adsorber catalysts

An *in situ* nano-Pt-edge XANES study was conducted on model Pt containing NOx adsorber catalysts. The catalyst used was ceria-supported platinum (1 wt%): samples were synthesized using the incipient-wetness-impregnation method and a platinum precursor. Powder samples were dispersed on the heater chips and assembled in the cell. XANES maps were extracted from the Pt-*L*_3_–*M*_5_ XRF signal collected at 150 energy points over the Pt-*L*_3_ absorption edge. The Pt-*L*_3_–*M*_5_ maps were generated by summation within a 200 eV window around the fluorescence peak. The Pt-*L*_3_ sequence was stacked and aligned to give the Pt-*L*_3_ XANES spectra at each pixel.

**Figure 7:**
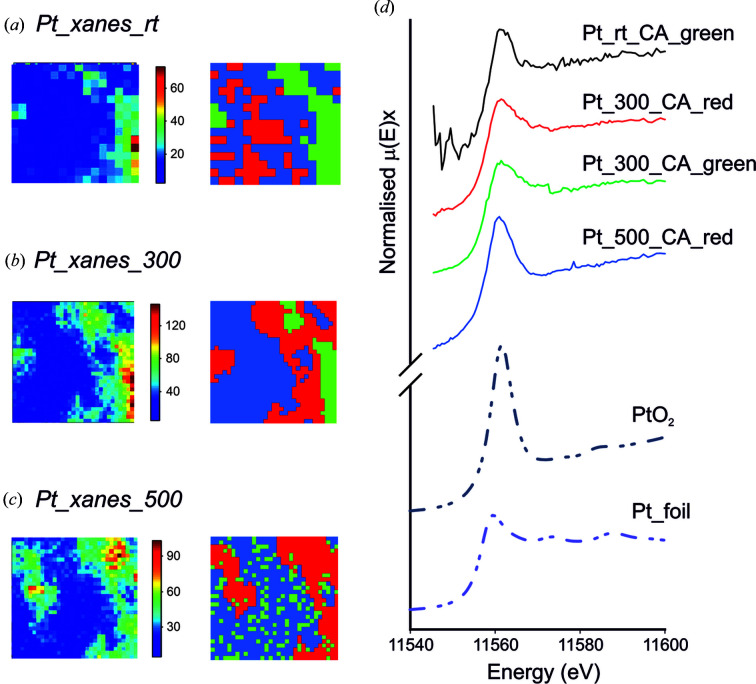
An Fe *K*α XRF map of the same area of the ceria-supported platinum catalysts under: (*a*) room temperature in an inert gas flow, (*b*) reducing conditions at 300°C and (*c*) re-oxidizing conditions at 500°C. XANES mapping was carried out on each area with the CA performed in *MANTiS*, grouping the pixels according to their spectroscopic variance shown on the right. (*d*) Individual XANES spectra extracted from selected CA on the samples in (*a*), (*b*) and (*c*), compared with the XANES spectra of two Pt standards: PtO_2_ and Pt foil.

Pt-*L*_3_ XANES maps were collected at (*a*) room temperature under inert gas flow (He, 1 bar), (*b*) at 300°C under reducing conditions (10% H_2_/He), and (*c*) at 500°C under re-oxidizing conditions (20% O_2_/He). The resulting spectra from the maps were analysed using principal component and cluster analysis (CA) in *MANTiS* (Lerotic *et al.*, 2014 ▸). The results are shown in Fig. 7 ▸. Inspection of the cluster spectra from conditions (*a*), (*b*) and (*c*), and comparison with the XANES spectra for Pt metal and PtO_2_ references by linear combination fitting (LCF), indicate the presence of 44 ± 9% Pt metal at room temperature (*a*). This is increased under the reducing conditions to an average of 68 ± 4% Pt metal, with small difference in spectra from the ‘green’ (57 ± 4%) and ‘red’ (78 ± 4%) clusters indicating some spatial variations (*b*). Under the oxidizing conditions (*c*), LCF indicates more oxidized Pt with an average Pt metal component of 29 ± 3%.

These results demonstrate the use of the *in situ* cell for beamline XRF and XANES mapping, opening the possibility of observing nanoscale spatial variation in catalyst activity under redox conditions using nanofocus X-ray microscopy. This setup can be further used to study more complex catalytic reactions and link observed changes such as oxidation state to catalytic activity and performance by connecting a mass spectrometer to the gas outlet line. Whilst there are challenges associated with these investigations (*e.g.* small sample volumes, the time resolution achievable, increased sample drift), careful experimental design, improved data-acquisition approaches (*e.g.* Quinn *et al.*, 2021*b* ▸) and the use of complementary techniques make nanoprobe *in situ* studies a vital tool.

## 5. Conclusions

*In situ* studies are key to understanding materials under practical conditions by process replication on a smaller scale. This understanding also requires a multi-scale approach such as linking formulation, pore structure, binder properties, particle size and chemistry. For X-ray microscopy, *in situ* measurements are critical to realizing new science, and many of the science cases for new low-emittance facilities are focused on exploiting flux gains for more dynamic studies. We have implemented, characterized and demonstrated an *in situ* cell for heating under gas flow that allows for the same sample and cell assembly to be used across an X-ray nanoprobe and a transmission electron microscope. We have successfully demonstrated variable temperature/gas-environment nano­scale XANES, with the design of the adaptor and the ease of transfer of the cell assembly between setups allowing for the possibility of correlative TEM measurements in the same heating and gas conditions. This opens exciting opportunities for complementary X-ray and electron microscopy techniques to be applied to a range of catalysts for understanding their properties *in situ*. The cell is available for use at the nanoprobe beamline (I14) at Diamond Light Source.
